# Supplementary material for: Vitamin D Modified DSS-Induced Colitis in Mice via STING Signaling Pathway
Source: Biology (Basel). 2025 Jun 18;14(6):715. doi: 10.3390/biology14060715 (PMC12190092; doi:10.3390/biology14060715)
Supplement: Supplementary file 1 [file biology-14-00715-s001.zip › Supplementary Materials S1: 16s-report of gut microbiota/Groups/diff_analysis/metagenomeRGBM/figure/boxplot/alllog.pdf]

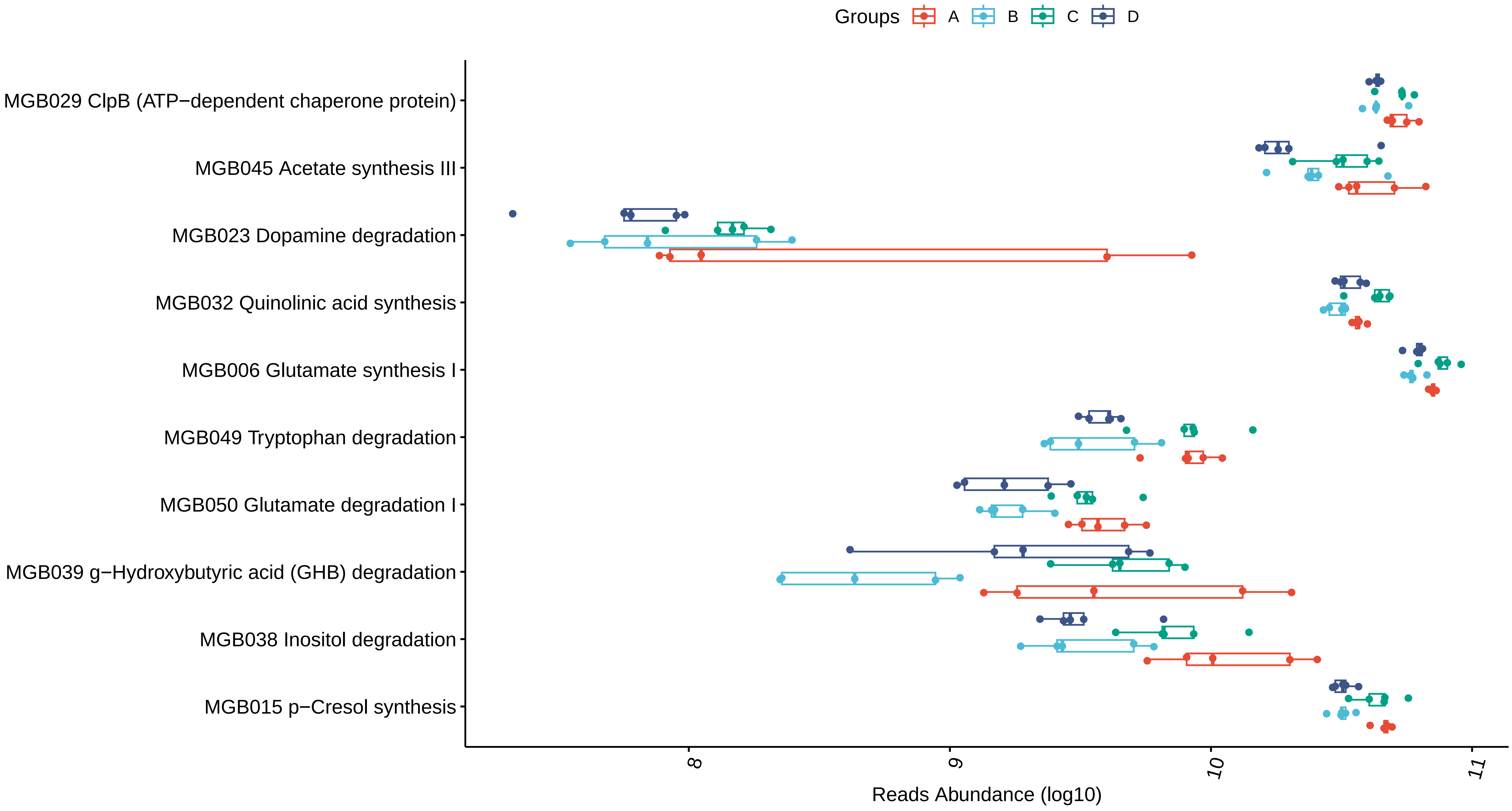
Groups
A
B
C
D
MGB029 ClpB (ATP−dependent chaperone protein)
MGB045 Acetate synthesis III
MGB023 Dopamine degradation
MGB032 Quinolinic acid synthesis
MGB006 Glutamate synthesis I
MGB049 Tryptophan degradation
MGB050 Glutamate degradation I
MGB039 g−Hydroxybutyric acid (GHB) degradation
MGB038 Inositol degradation
MGB015 p−Cresol synthesis
8
9
10
11
Reads Abundance (log10)
